# Characterization of Relevant Biomarkers for the Diagnosis of Food Allergies: An Overview of the 2S Albumin Family

**DOI:** 10.3390/foods10061235

**Published:** 2021-05-29

**Authors:** Cristina Bueno-Díaz, Laura Martín-Pedraza, Jorge Parrón, Javier Cuesta-Herranz, Beatriz Cabanillas, Carlos Pastor-Vargas, Eva Batanero, Mayte Villalba

**Affiliations:** 1 Department of Biochemistry and Molecular Biology, Complutense University of Madrid, 28040 Madrid, Spain; crbueno@ucm.es (C.B.-D.); jparron@ucm.es (J.P.); cpasto01@ucm.es (C.P.-V.); ebataner@ucm.es (E.B.); 2 RETIC ARADyAL, Health Research Institute Carlos III, 28029 Madrid, Spain; lauramp627@gmail.com (L.M.-P.); j.cuestaherranz@gmail.com (J.C.-H.); 3 Molecular Allergology Group, Paul-Ehrlich Institut, 63225 Langen, Germany; 4 Health Research Institute Fundación Jiménez Díaz (IIS-FJD, UAM), University Hospital Fundación Jiménez Díaz, 28040 Madrid, Spain; 5 Department of Allergy, Research Institute Hospital 12 de Octubre, 28041 Madrid, Spain; beatriz.cabanillas@salud.madrid.org

**Keywords:** food allergy, 2S albumin, biochemical characterization, cross-reactivity, anaphylaxis

## Abstract

2S albumins are relevant and often major allergens from several tree nuts and seeds, affecting mainly children and young people. The present study aims to assess how the structural features of 2S albumins could affect their immunogenic capacity, which is essential to comprehend the role of these proteins in food allergy. For this purpose, twelve 2S albumins were isolated from their respective extracts by chromatographic methods and identified by MALDI-TOF mass-spectrometry. Their molecular and structural characterization was conducted by electrophoretic, spectroscopic and in silico methods, showing that these are small proteins that comprise a wide range of isoelectric points, displaying a general high structure stability to thermal treatment. Despite low amino acid sequence identity, these proteins share structural features, pointing conformational epitopes to explain cross-reactivity between them. Immunoblotting with allergic patients’ sera revealed those possible correlations between evolutionarily distant 2S albumins from different sources. The availability of a well-characterized panel of 2S albumins from plant-derived sources allowed establishing correlations between their structural features and their allergenic potential, including their role in cross-reactivity processes.

## 1. Introduction

Foods represent one of the greatest antigenic loads the human immune system must face, tolerance being the normal physiological response. However, the prevalence of allergic reactions against foods has increased in recent decades, being estimated that 3–4% of the adult population and nearly 5% of children in Western countries suffer any kind of food allergy [1]. Among potential allergenic foods, a significant percentage corresponds to those from plant sources, highlighting the contribution of tree nuts, spices, seeds and fruits since hypersensitivity reactions against them begin at early ages and often persist throughout the life of individuals [2]. These foods are also known for their benefits to human health, providing a large quantity of beneficial compounds, some of them not available in animal origin food. Moreover, they are not only additional components in meals or snacks, but also oils are obtained from them for their use in food and cosmetic industries.

Currently, the development of new tools for food allergy diagnosis and management includes the employment of single isolated well-characterized allergens to identify those able to bind specific IgE (sIgE) from the patients’ serum. These diagnostic approaches, also known as component resolved diagnosis (CRD), seek for the identification of potential allergenic sources and for the establishment of a correlation between sensitization patterns of patients and the symptoms displayed, in order to obtain a personalized diagnosis [3]. Therefore, the isolation and characterization of food allergens from a biochemical and immunological point of view are highly relevant for their potential use in diagnosis, prevention and treatment of hypersensitivity reactions against foods.

The seed storage proteins are the most abundant allergens from plant-derived sources since they constitute nearly 70% of the total protein content in seeds [4]. In general, storage proteins are synthetized at high levels in specific tissues and their primary structures contain high proportions of methionine and cysteine, being a great source of nutrients for the plant during germination. The sensitization to these proteins is related to the development of severe reactions, including gastrointestinal ones or even anaphylaxis [5]. Their structural characteristics may explain their allergenic potential, making them interesting candidates for new clinical approaches like immunotherapy or CRD.

Among seed storage proteins, 2S albumins are considered relevant allergens from several plant-derived foods, affecting mainly to both children and the young people [6]. These are usually heterodimeric proteins whose structure is stabilized by disulfide bonds established through a highly conserved Cys pattern, providing them with high resistance to thermal and enzymatic treatments [7]. Consequently, these proteins are believed to resist food processing and digestion, reaching the intestinal lumen practically intact where they would interact with the immune system associated to the gut and initiating an allergic response. Their structural resemblance at three-dimensional (3D) level contrasts with the low conservation of their amino-acid sequence, which may have implications in patients’ sensitization profiles since it can involve cosensitization and cross-reactivity processes [8]. 

Most of the 2S albumins have been described as major allergens from their respective natural sources. They have also been considered markers of sensitization and potential triggers of anaphylaxis [9,10]. Their immunological relevance makes them suitable candidates for their implementation in precision diagnostic technologies. However, there is a lack of consistent studies regarding 2S albumins’ structural features and their implications neither sensitization patterns displayed by allergic patients nor their potential cross-reactivity. In the present work, we aimed to explore the structural characteristics of 2S albumins and their link with clinical manifestations displayed by allergic patients; moreover, we set out to highlight the cross-reactive potential of these allergens due to the availability of a wide panel of well-defined 2S albumins from several plant sources.

## 2. Materials and Methods

### 2.1. 2S Albumins Isolation and Identification

**Figure A1:**
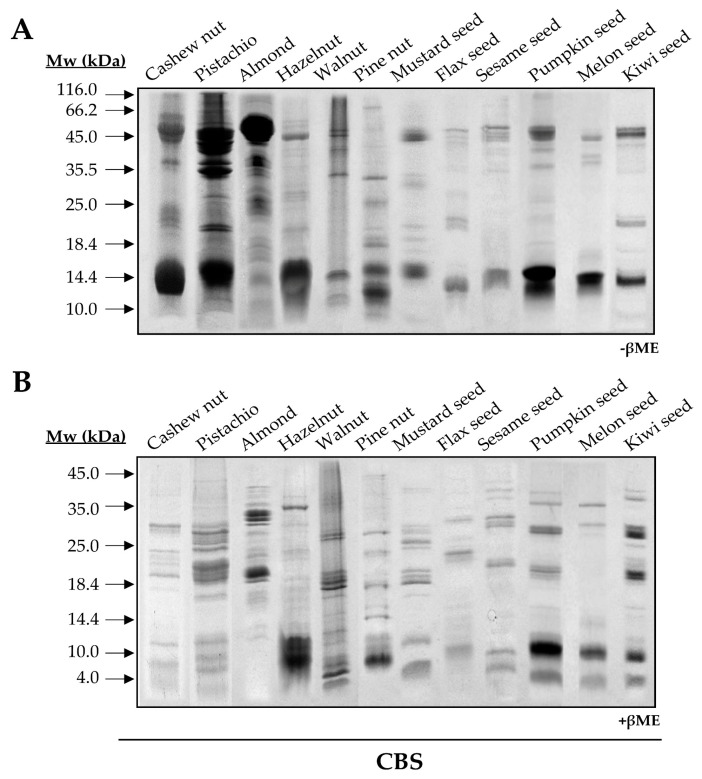
SDS-PAGE profiles of total protein extracts from plant-derived sources. Electrophoresis were conducted under non-reducing (−βME) (**A**) and reducing (+βME) (**B**) conditions, last one by treating samples with reducing agent βME for 10 min at 90 °C.

Total protein extracts from tree nuts and seeds were obtained as previously described [11]. Protein profiles of each extract were analyzed in 17% SDS-PAGE (Figure A1).

2S albumins were isolated by means of two chromatographic steps: first, a size exclusion chromatography using a Sephadex G-50 Medium column equilibrated with 0.15 M ammonium bicarbonate pH 8.0 at a flow rate of 3 mL/min. Second, a reverse phase-high performance liquid chromatography (RP-HPLC), using a C-18 reverse phase column, with an acetonitrile gradient from 0% to 60%. For Sin a 1 isolation, a SP-Sephadex C-25 ion exchange column was employed, in a 3–50 mM sodium pyrophosphate gradient. Once proteins were isolated, they were resuspended in 20 mM ammonium bicarbonate and stored at −20 °C. Protein concentration was determined by absorption spectroscopy, using a theoretical extinction coefficient (ε) for each protein, or by BCA method (Micro BCA protein assay kit, Thermo Scientific, Rockford, IL, USA) for those proteins whose theoretical ε was not available. Ara h 2 used for these experiments was purchased from Indoor Biotechnologies and although was included in the immunologic assays, it was not shown in the electrophoretic analysis.

### 2.2. Electrophoretic Procedures for Protein Characterization

Extracts quality, protein purity and apparent molecular mass were monitored by SDS-PAGE using Mini-Protean II (Bio-Rad) systems with separating 17% polyacrylamide gels. Coomassie Brilliant Blue R-250 (Merk, Darmstadt, Germany) was employed for staining (CBS). Precision Plus Protein TM All Blue (Bio-Rad, Hercules, CA, USA) molecular mass leaders were used as reference.

Two-dimensional-electrophoresis (2D SDS-PAGE) was performed with purified proteins (10 µg) to analyze their isoelectric points (pI) and further detection by CBS, employing for the first dimension the ReadyPrep™ 2D starter kit (Bio-Rad) and IPG strips (Bio-Rad, Hercules, CA, USA) pH 3–10 gradient. The second dimension was carried out in an SDS-PAGE as described above.

### 2.3. Circular Dichroism

Circular dichroism (CD) studies were performed on a Jasco J-715 spectropolarimeter (Japan Spectroscopic Co., Tokyo, Japan) equipped with a CDF-426S Peltier temperature-control system interfaced with a NESLAB RTE111 water bath. Far-UV CD spectra were recorded at 20 and 85 °C in a 0.1 cm-pathlength quartz cuvette (200 µL) (Hellma, Baden-Wuerttemberg, Germany) at protein concentrations of 0.2–0.5 µg/µL. All samples were solved in 20 mM ammonium bicarbonate, pH 8.0. Spectra were recorded at 50 nm/min, and control buffer baseline was subtracted. Thermal denaturation was monitored by recording the [*θ*]_220_ (molar ellipticity at a fixed wavelength of 220 nm) while heating or cooling at 1 °C/min. 

The results are expressed as mean residue weight (MRW) molar ellipticity:

(1) [θ]MRW =θ×MC×l×10

where *θ* is the observed ellipticity, *M* is the average molecular weight per residue, *C* is protein concentration (mg/mL) and *l* optical pathlength (cm).

### 2.4. Computational Tools for the Analysis of 2S Albumins Structural Features

Signal peptide prediction: Signal P 5.0 (ExPASy) online program based on artificial neuronal networks (http://www.cbs.dtu.dk/services/SignalP). Sequence alignment: proteins sequences were obtained from NCBI Protein or UniProt databases. Sequences alignment was performed using ClustalW program (https://www.ebi.ac.uk/Tools/msa/clustalo). Tertiary structure prediction and representation: automatic modelling of proteins using Swiss-Model tool (ExPASy) based on protein sequence similarity (https://swissmodel.expasy.org/). Brazil nut 2S albumin, Ber e 1, was employed as the template (2LVF, PDB).

### 2.5. Western Blotting with Patients’ Sera 

Sera from allergic patients were used for testing and characterizing the potential allergenicity. Patients were recruited from Allergy Service of five Spanish hospitals from Madrid. Written informed consent was obtained from all patients. The work was performed accomplishing the Ethic Guidelines of Complutense University of Madrid

Pools for immunoassays were composed by four patients sera, whose specific clinical features were previously described in works of the group [12,13,14]. In general, they reported food allergy against a single source, displaying, positive skin prick test (SPT, bump diameter >3 mm) and/or sIgE levels (>1 kU/L) determined by ImmunoCAP (Thermo Scientific, Rockford, IL, USA). Patients sera were pooled together according to the source against which they described food allergy: peanut, cashew nut, hazelnut, mustard seed, pine nut, pumpkin seed or flaxseed. Sera from non-atopic individuals were employed as negative control for immunoassays.

Purified proteins (2 µg) were blotted onto nitrocellulose membranes (GE Healthcare Life Sciences, Marlborough, MA, USA) after SDS-PAGE. Immunodetection of proteins was performed, by using individual or an equivolumetric pool of two patients sera, in both cases diluted 1:5 in 3% skim milk powder (SMP) with PBS containing 0.1% Tween20 (PBS-T). 

Binding of human IgE was detected with mouse anti-human IgE monoclonal antibody (1:5000 diluted) kindly provided by ALK-Abelló (Madrid, Spain), followed by horseradish peroxidase-labelled rabbit anti-mouse IgG polyclonal antibody (1:2000 diluted; Pierce, Rockford, IL, USA). The chemiluminescent signal was developed by ECL-Western blotting reagent and detected in a luminescent imager analyzer LAS3000 (Fujifilm, Tokio, Japan). Quantitation of the signal was performed in triplicate using the computer program Multigauge V3.0 (Fujifilm, Tokio, Japan).

## 3. Results

### 3.1. Experimental Characterization of Isolated 2S Albumins

#### 3.1.1. The Polymorphic Nature of 2S Albumins

**Figure 1:**
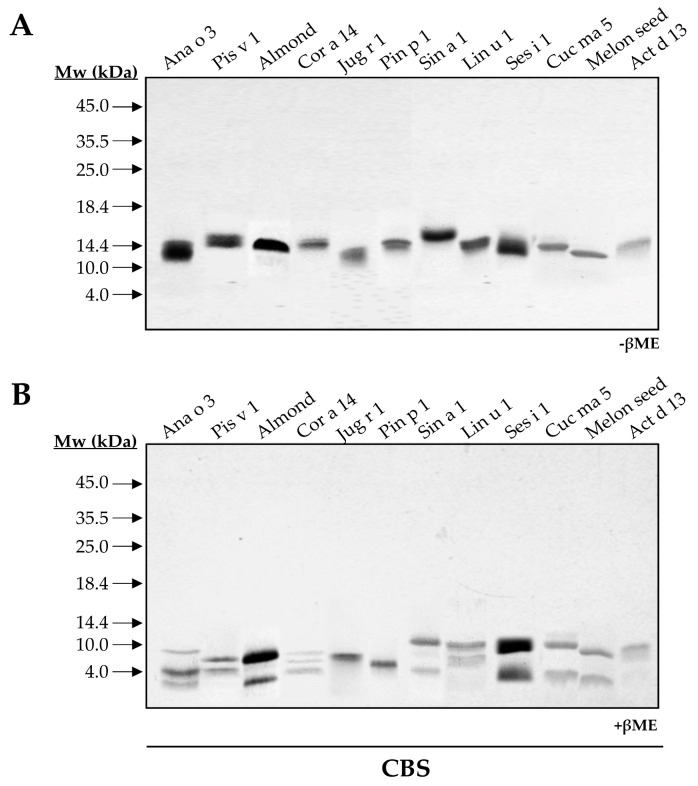
SDS-PAGE of purified 2S albumins from plant-derived sources. Electrophoresis was conducted under non-reducing (**A**) and reducing conditions using βME (**B**). Molecular mass markers (Mw) are indicated with arrows. CBS: Coomassie Blue Staining.

To elucidate and compare the structural features of the 2S albumin family, twelve 2S albumins were obtained from their respective sources: Ana o 3 (cashew nut), Pis v 1 (pistachio nut), almond 2S albumin, Cor a 14 (hazelnut), Jug r 1 (walnut), Pin p 1 (pine nut), Sin a 1 (mustard seed), Lin u 1 (flaxseed), Ses i 1 (sesame seed), Cuc ma 5 (pumpkin seed), melon 2S albumin and Act d 13 (kiwi seed). SDS-PAGE of all isolated proteins showed low molecular masses ranging from 12 to 15 kDa (Figure 1A). In the presence of the reducing agent βME, ten out of twelve 2S albumins were split into two polypeptide chains: a large chain around 8–10 kDa and a small one around 3–5 kDa, a common feature attributed to these allergens (Figure 1B).

As expected, walnut and pine nut albumins, Jug r 1 and Pin p 1, displayed a single polypeptide chain under reducing conditions as previously reported [15,16]. However, the loss of intrachain disulfide bridges in reducing conditions modified their electrophoretic mobilities. Moreover, this behavior contrasts with that observed for Ana o 3 and Cor a 14, which exhibited three polypeptide chains in their structures (Figure 1B). These results may indicate the presence of isoforms for these proteins due to different proteolytic processing of 2S albumins in the seed. 

**Figure 2:**
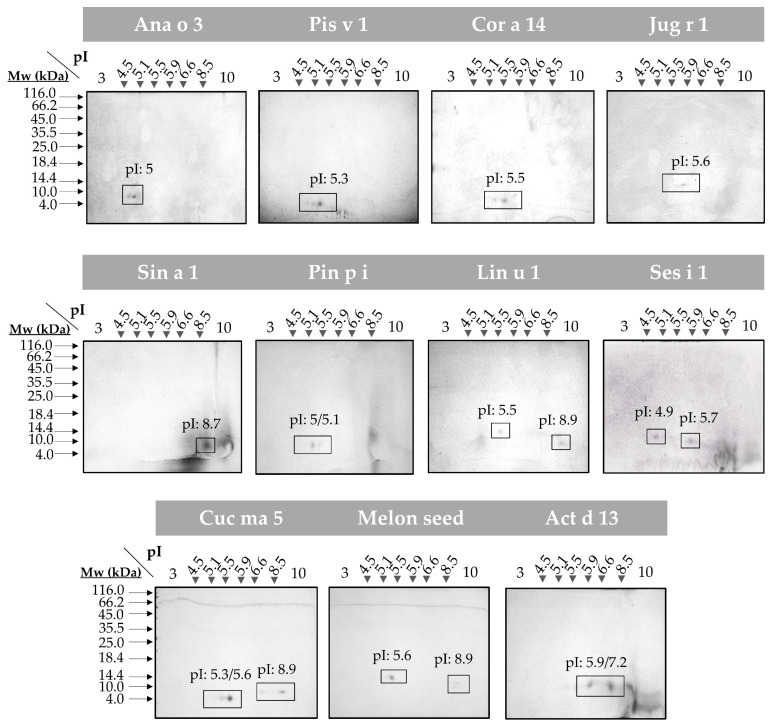
2D SDS-PAGE of the isolated 2S albumins. Spots corresponding to isolate protein polypeptide chain isoforms are boxed. Isoelectric points (pI) of each isoform are indicated. Molecular mass markers (Mw) are indicated with arrows.

To evaluate the presence of isoforms in all the 2S albumins, 2D SDS-PAGE were assayed (Figure 2). Broad isoelectric points (pI) differences were detected in all proteins considering the great variability of their primary structures. Moreover, the polymorphic nature of these 2S albumins was elucidated (e.g., Ana o 3, Cor a 14 and Cuc ma 5) as indicated by the polypeptide chain isoforms (number of spots) that displayed similar molecular masses but different pI values.

#### 3.1.2. Structural Behavior of 2S Albumins to Thermal Treatment

**Figure 3:**
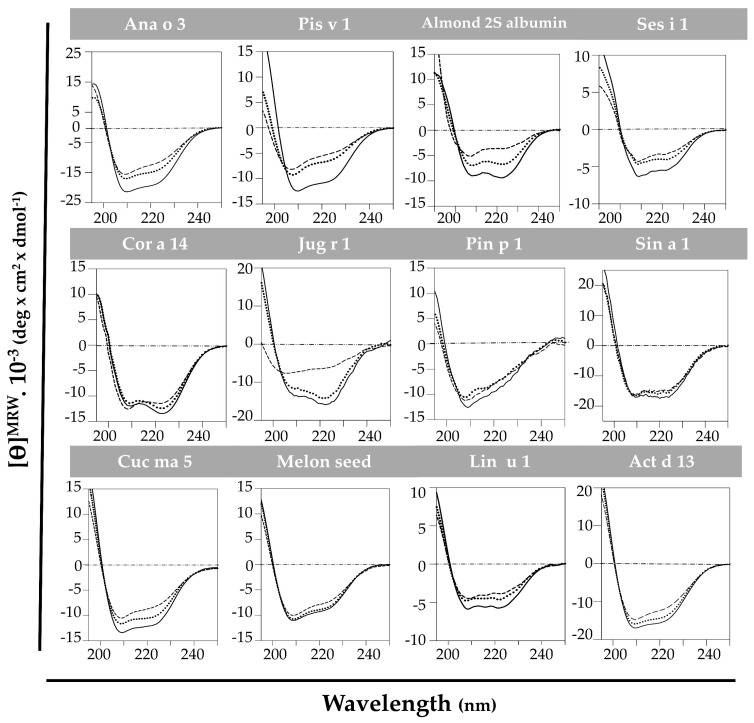
Spectroscopic characterization by circular dichroism at far-UV of 2S albumins. Spectra were measured at 20 °C (---), at 85 °C (----) and cooling down again at 20 °C (····).

The high structural stability against thermal treatment attributed to the 2S albumin family was analyzed by spectroscopic assays. CD at far-UV of the purified proteins brought up that this behavior was not as generalized as expected (Figure 3). All 2S albumins exhibited a well-folded structure mainly composed of α-helical motives. Pin p 1 exhibited higher random coil contribution than the others. Nevertheless, most of the proteins presented a partial loss of their initial structure when heated at 85 °C. In general, the increase in temperature affected the global structure of these proteins. The reduction of ellipticity and displacement of CD spectra’ minimum indicate an increment on random coil motives contribution. Importantly, only Sin a 1, melon seed 2S albumin and Pin p 1 retained their original structures after heating at 85 °C, indicating their structural stability under thermal processing.

When cooling back to 20 °C, only Cor a 14 and Act d 13 completely recovered their initial structures, while 2S albumins from almond and Cuc ma 5 did it partially. It is remarkable that Jug r 1 was able to denature and completely renature after heat treatment. As shown in Figure 3, walnut 2S albumin ellipticity dramatically decreased when heating at 85 °C shifting the CD spectrum to the left, indicating an increment in the contribution of random conformation. When cooling back to 20 °C, the protein fully recovered its initial structure. 

Pis v 1, Ana o 3, Ses i 1 and Lin u 1 do not completely loose the structure after the heat treatment but they do not recover their initial structures after cooling down. A possible aggregation of these proteins has been suggested at high temperatures, as reported for Ara h 2 [17]. 

### 3.2. In Silico Analysis of the 2S Albumin Structures

2S albumin primary and tertiary structures were analyzed by computational methods, employing bioinformatic tools for sequence alignment (GeneDoc) and prediction of 3D structure (Swiss-Prot). Signal peptides determined with SignalP online tool were not considered in these analyses.

**Figure 4:**
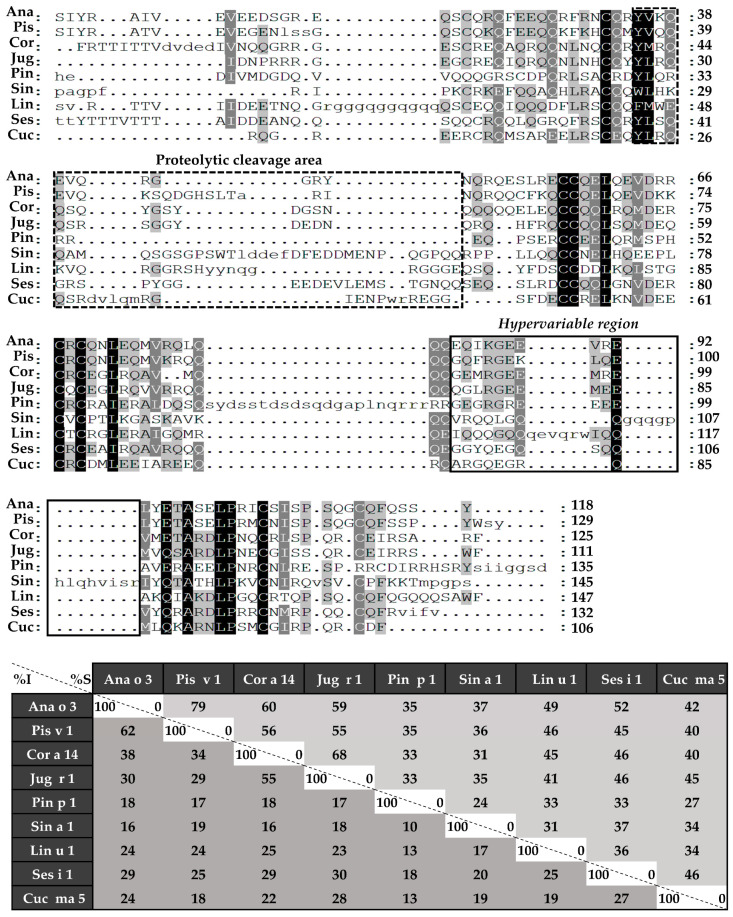
Sequence alignment of isolated 2S albumins. Primary structures of 2S albumins were aligned with ClustalW online tool. The table under alignment shows percentages of identity (% I, dark grey) and similarity (% S, light grey) between the different proteins. Discontinuous line boxes indicate the proteolytic cleavage area between light and heavy chains, while continuous line boxes indicate the hypervariable region.

**Figure 5:**
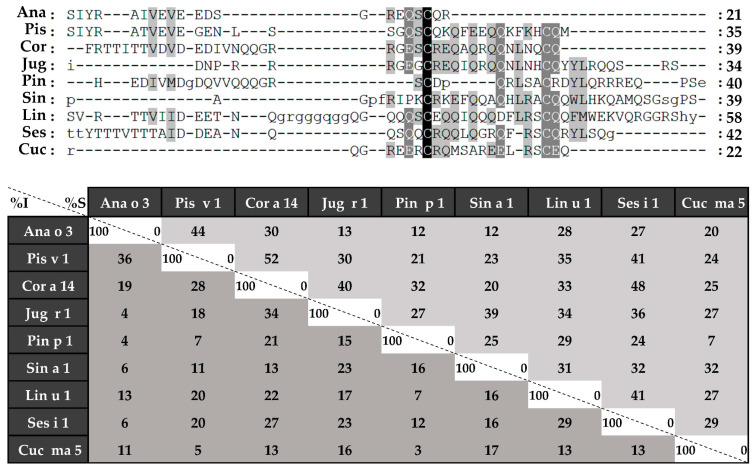
Amino acid sequence alignment of isolated 2S albumins light chains. The table under alignment shows percentages of identity (% I, dark grey) and similarity (% S, light grey).

**Figure 6:**
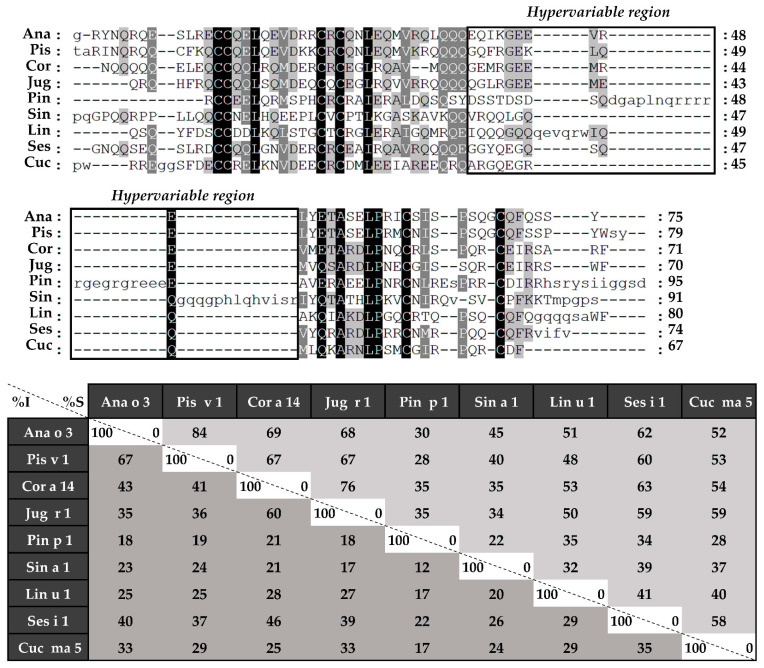
Amino acid sequence alignment of isolated 2S albumins heavy chains. The table under alignment shows percentages of identity (% I, dark grey) and similarity (% S, light grey). Continuous line boxes indicate the hypervariable region.

Sequence alignment was performed in the first place with complete amino acid sequences (Figure 4), followed by those of light (Figure 5) and heavy chains (Figure 6), separately. 2S albumins are encoded by multigene families, with a complex processing, which leads to different sequences with a low similarity among biological sources [7,8]. As detailed in Figure 4, protein sequences showed identity degrees oscillating around 18–39%. Only Pis v 1 and Ana o 3 from pistachio and cashew nut, reached values of around 62%. In general, despite the cysteine pattern and the regions spanning it, the remaining sequence seems to have a low sequence identity degree.

Considering the alignment of the light chains (Figure 5), identity percentages were below 40%, even when comparing those chains from phylogenetically related sources, like Ana o 3 and Pis v 1. However, when heavy chain alignment was analyzed (Figure 6), identity percentages up to 67% were observed in the case of Ana o 3 and Pis v 1 and up to 60% in the case of Jug r 1 and Cor a 14. Nevertheless, inside heavy chains there is a segment that exhibits low similarities between 2S albumins known as the “hypervariable region”, which is located between the fourth and fifth Cys residues.

**Figure 7:**
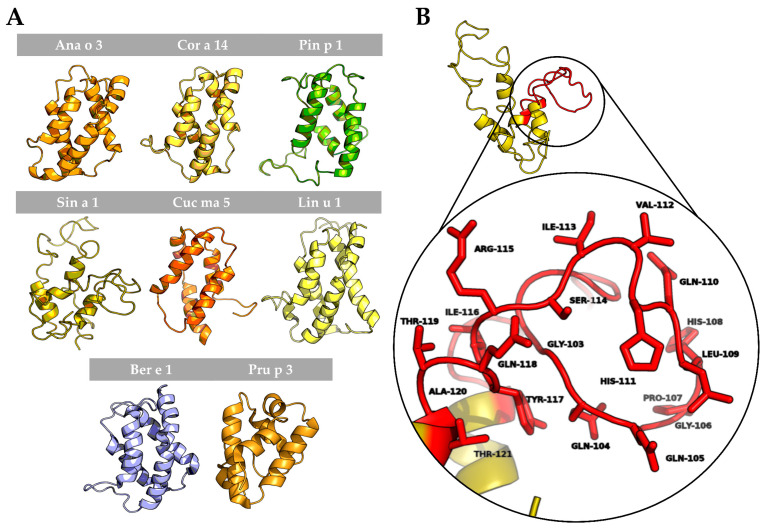
In silico modeling of 2S albumins three-dimensional structure. (**A**) Three-dimensional modeling of isolated 2S albumins, using the annotated structure of Ber e 1 (Brazil nut 2S albumin) as the template. nsLTP from peach, Pru p 3, has been included for comparative purposes. (**B**) Hypervariable region from mustard seed allergen, Sin a 1, and zoom into one of its allergenic epitopes located in this region.

To better understand the possible structural similarities among 2S albumins, 3D structures of these proteins were studied (Figure 7A). 2S Albumins were characterized by a compact bundle of 4–5 α-helices with a C-terminal loop. The high similarity between the 3D structures of these proteins might indicate the presence of conserved structural epitopes between them [18], even in poorly preserved amino acid sequences.

In addition to the structural epitopes, the *hypervariable region*, which contains some of the most immunogenic epitopes described for these proteins [7,8], is located in an exposed loop between the fourth and fifth helix (Figure 7B), facilitating the access of the immune system to the epitopes located in this area. In conclusion, despite the general low resemblance between 2S albumins primary structure, the presence of some preserved epitopes at sequential and structural levels indicates the cross-reactive potential of these proteins even in non-related sources.

### 3.3. The Link between the Structure and the Allergenicity of 2S Albumins

**Figure 8:**
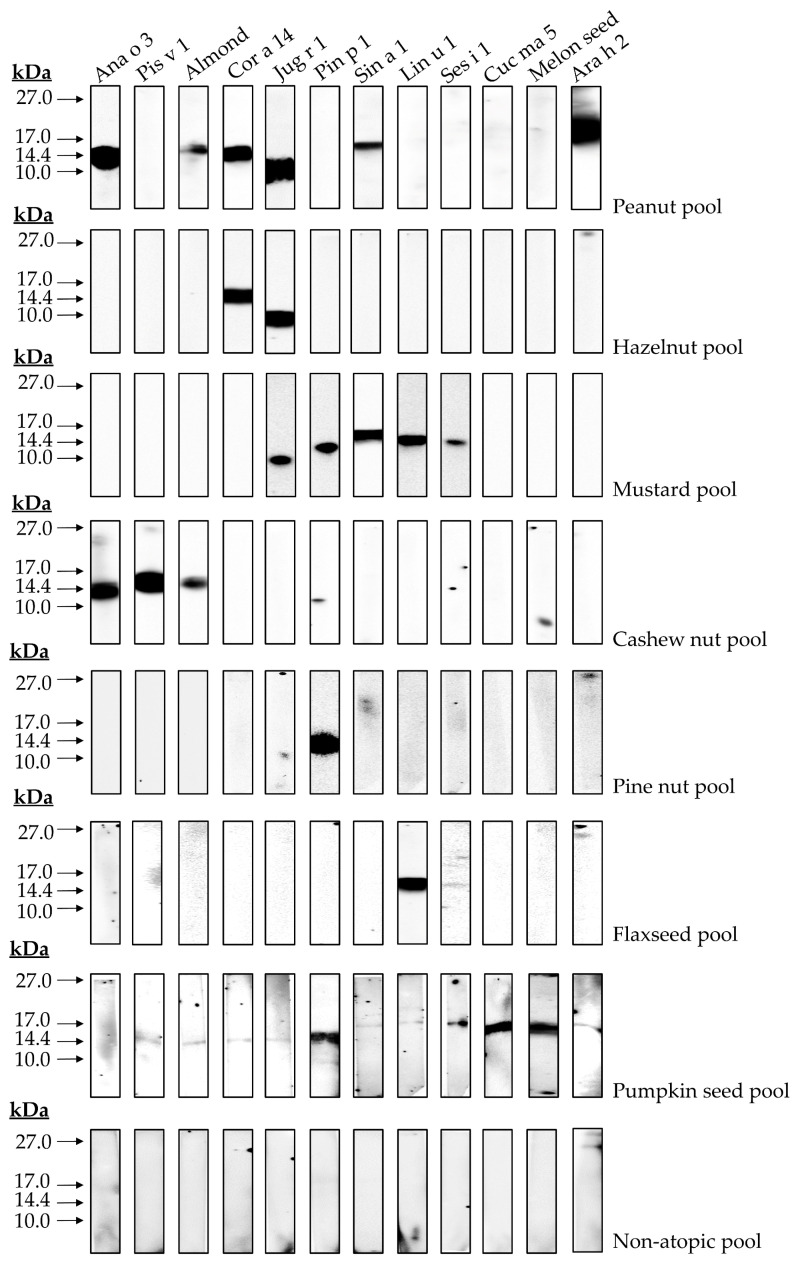
Immune detection of isolated 2S albumins tested in blood serum of different patient cohorts. A pool of non-atopic patients was employed as control.

Immunological assays employing allergic patients’ sera may reveal possible correlations between structural characteristics and immunogenicity displayed by 2S albumins. In the preliminary analysis showed in Figure 8, possible protein clusters recognized by the pools of patients sensitized to 2S albumins were detected. Firstly, the exclusive recognition of the 2S albumins from pine nut and flaxseed in their respective cohort of patients was observed. Other groups of patients reacted to at least two or more different sources. In the case of hazelnut, only two proteins were recognized by this cohort of patients, Cor a 14 and Jug r 1. This suggests a possible cross-reactivity between these two nuts since both proteins are phylogenetically related and they show a higher sequence identity as mentioned previously, suggesting the presence of shared epitopes in both proteins. Similar findings were obtained between cashew nut and pistachio, both Anacardiaceae members, showing Ana o 3 and Pis v 1 in the corresponding patient serum cohort.

Moreover, patients allergic to peanut, mustard seed and pumpkin seed showed, not only their respective 2S albumins, but also those from other non-related sources, such as almond, hazelnut, walnut, mustard seed and melon seeds albumins. Similarly, patients allergic to mustard seeds through Sin a 1 reacted to walnut, pine nut, flaxseed and sesame seed albumins. Previous data reported by our group showed this cross-reactivity between pumpkin seed or mustard seed and pine nut through their 2S albumins [12,14], although identity between both allergens was below 50%. These results, in combination with those here exposed, suggest a wider cross-reactivity potential of 2S albumins than previously thought.

## 4. Discussion

The study of the structural properties involved in the allergenicity of certain proteins has been the focus of research in the last decades [8]. 2S albumins stand out as major allergens in several plant-based foods widely consumed by the population. Several studies have reported the impact of 2S albumins in the development of severe systemic symptoms in allergic patients [19,20]. The present work describes the obtention for the first time of twelve allergenic 2S albumins from different foods, some of them recently introduced in our diet, such as melon or pumpkin seeds. All of them have been isolated by similar procedures with minor modifications and fully characterized. This comparative study at the structural and immunological level has seeded light to understand the relation between the structural characteristics of these proteins and their impact in food allergy [21].

2S albumins are included in the low molecular mass proteins fraction (below 20 kDa) (Figure A1). Their abundance in seeds and nuts has been linked to an increased risk of allergic sensitization, as some of these proteins account for almost 30% of the total content [22,23]. 2S albumins belong to multigene families and undergo different proteolytic maturation, resulting in different isoforms, which contribute to their higher proportion relative to other seed proteins. Here, we demonstrated the presence of isoforms in Ana o 3, Cor a 14 and Cuc ma 5 proteins, as they split into more than two polypeptide chains with different length when treating them with reducing agents in both mono and 2D-electrophoresis.

The disulfide bridges found in 2S albumins provide a compact structure that is stable to heat and enzymatic treatments [16,24]. In these terms we found that Sin a 1, melon seed 2S albumin and Pin p 1 were not affected by heating and retained their native structures. However, CD spectra revealed that most of the proteins suffered a partial loss of their native structure when heating at 85 °C, and only some of them recovered it after the initial conditions were restored such as Jug r 1, Act d 13 and Cor a 14. Moreover, Ana o 3, Pis v 1 and Ses i 1 did not recover their initial conformations when cooling back to 20 °C. This behavior was previously described for other 2S albumins, attributing to protein aggregation at high temperatures this loss of structure and therefore affecting their allergenicity, as reported for Ara h 2 by Starkl et al. [25].

Some food processing steps in industry require high temperatures. It is believed that most of the epitopes presented in the 2S albumins are not affected due to their structural stability and therefore, are accessible for immune system recognition. However, allergens respond differently depending on the food processing step to which they are submitted [26]. Thermal treatments may lead to chemical modifications of allergens in the presence of other compounds of the food matrix (e.g., carbohydrates, lipids, etc.), altering crucial epitopes or creating new ones. Under this scenario, most of the studies have been done in peanut allergens [27,28], showing that Ara h 2 allergenicity capacity increases after the glycation of certain residues through the Maillard reaction [29]. Future studies should assess chemical modifications in other ubiquitous sources, like spices or seeds (e.g., mustard or sesame seeds), thus it would provide very valuable information about proteins’ allergenicity, which may be enhanced or reduced by either food processing or components present in the mixture [30], making some products safer or riskier for allergic patients.

Computational tools have helped to deep in the structural characteristics of proteins and the implications for their allergenicity [31]. Amino acid sequence alignment revealed that only residues surrounding the cysteine patterns exhibit higher identities when comparing with the whole sequence. This may be due to the structural role of these residues since they are involved in the maintenance of the tertiary structure of 2S albumins [32].

The *hypervariable region*, located in the most exposed area of these proteins, presents the lowest identity values and the most immunogenic epitopes that have been described in of some the 2S albumins [8,33] (Figure 7B). Due to its low sequence preservation, it has been believed that those epitopes are not involved in the cross-reactivity but polysensitization of atopic patients [8]. Only phylogenetically related allergens exhibit higher similarities in this area, as it is the case of *Brassicaceae* 2S albumins [34,35] or the *Anacardiaceae* members [13].

Unlike the primary structure, the 3D conformation seems to be highly conserved across 2S albumins from different sources. Recent studies have predicted and analyzed already described structural epitopes from several 2S albumins [36], highlighting Ara h 2 due to its potential cross-reactivity with albumins from tree nuts and sesame seeds and its clinical implications in food allergy.

The structural conformation displayed by 2S albumins is similar to other proteins also described as food allergens, the nsLTPs. However, they exhibit a more conserved amino acid sequence and are considered panallergens that are involved in cross-reactivity processes [37]. Moreover, their ability to binding lipids confers them the protection against digestion [38]. The capacity of some 2S albumins to interact with lipids has been previously described [39], although the presence of a specific lipid-binding cavity in these allergens has not been clearly demonstrated [40,41]. In the case of Ber e 1, the major allergen and 2S albumin from Brazil nut, its interaction with lipids has been described as necessary for the oral sensitization against this allergen and for its protection against enzymatic digestion in murine models [41,42]. It is required more evidences to understand how lipid interactions affect to the 2S albumins allergenicity.

Finally, the immunological assays with blood serum from allergic patients revealed the different possible scenarios in food allergy mediated by 2S albumins. Patients allergic to plant-derived foods often suffer from hypersensitivity reactions from several sources. There are two possible explanations: first, a multiple and independent primary sensitization to several 2S albumins from different sources, with no immunological correlations between them. Additionally, second, a potential cross-reactivity process among albumins clusters even if they are evolutionary distant could occur. The presence of preserved epitopes among both proteins has been recently reported [43]. Potential cross-reactivity of these allergens and its implications at the clinical level has been reported by our group [13], describing for the first time a pistachio-cashew nut allergic syndrome in a well-characterized group of patients whose severe symptomatology was related to the sensitization to *Anacardiaceae* 2S albumins and the cross-reactivity processes between them. Recent information regarding 2S albumins structure has pointed out the presence of preserved epitopes among non-related sources. Epitope mapping of Pin p 1 revealed shared epitopes with albumins from peanut, sesame seed and hazelnut, among others [44], while studies conducted with allergic patients showed Pin p 1 cross-reaction with Sin a 1 [12] and Cuc ma 5 [14]. These results support the existence of conserved epitopes in 2S albumins. Their implications in clinical manifestations displayed by a larger cohort of patients will provide useful information that can be employed at the clinical level to improve patients’ diagnosis and management. Future studies should evaluate if in vitro results can be extrapolated to clinical manifestations of patients, or if this cross-reactivity among 2S albumins remains experimental but not of clinical relevance.

## 5. Conclusions

The availability of a panel of highly purified food allergens has allowed their structural characterization employing biochemical, spectroscopic, immunological and in silico techniques. The results extracted from those analyses have supposed a step forward in the comprehension of how protein structural features are involved in their allergenic capacity: abundance, polymorphism, structure stability and epitopes disposition and preservation. Immunological assays with small allergic patients’ cohorts have allowed one to establish the potential allergenic associations between the 2S albumins from different sources. Although this work brings together for first time the structural and immunological features of twelve allergens from the same family, additional clinical studies with broader allergic populations are still needed to corroborate the clinical relevance of their cross-reactivity potential. Nevertheless, the present work highlights the importance of 2S albumins as markers of food allergy, not being restricted only to genuine sensitization but also to potential cross-reactivity processes. The employment of these proteins in molecular diagnosis approaches like component resolved diagnosis may improve patients’ characterization, diagnosis and treatment.

## Data Availability

The data presented in this study are available on request from the corresponding author.
